# Genetic diversity of respiratory enteroviruses and rhinoviruses in febrile adults, Singapore, 2007‐2013

**DOI:** 10.1111/irv.12662

**Published:** 2019-09-30

**Authors:** Martin Linster, Celeste Donato, Marcus G. Mah, Miguel L. Grau, Jenny G. Low, Eng Eong Ooi, Yvonne C.F. Su, Gavin J.D. Smith, Dhanasekaran Vijaykrishna

**Affiliations:** ^1^ Programme in Emerging Infectious Diseases Duke‐NUS Medical School Singapore; ^2^ Department of Microbiology, Biomedicine Discovery Institute Monash University Clayton Victoria Australia; ^3^ Duke Global Health Institute Duke University Durham North Carolina

**Keywords:** enterovirus, febrile illness, phylogenetic analysis, rhinovirus, serotypes, Singapore, virus diversity

## Abstract

To understand the genetic diversity and patterns of circulation of rhinoviruses (RV) and enteroviruses (EV) in Singapore, we retrospectively screened 2950 nasal swab samples collected from adults presenting to primary care services with signs of febrile illness in Singapore during 2007‐2013 using sequencing and phylogenetic methods. Through sequencing and phylogenetic analysis, our results show the year‐round circulation of the three rhinovirus species, A, B, and C. A diverse set of RV/EV serotypes were detected in Singapore with a predominance of RV‐A in all years, whereas serotypes EV‐C A21 and EV‐D68 were only sporadically detected. This study highlights the previously unrecognized diversity and burden in the adult population in Singapore.

## 1 INTRODUCTION

Enteroviruses (EV) and rhinoviruses (RV) are a diverse group of single‐stranded RNA viruses subdivided into 15 species within the genus *Enterovirus* (EV‐A to K and RV‐A to C) and belonging to the family *Picornaviridae*. In excess of 100 EV and 150 RV, serotypes have been described from humans, with considerable differences in virulence, and exhibit a particularly broad range of clinical infections, ranging from subclinical infections to febrile illness, sore throat and rash (hand, foot, and mouth disease [HFMD]), meningitis, and paralysis (poliomyelitis).^1^ The majority of EV serotypes is associated with gastroenteric infections that are transmitted via the oro‐fecal route. EV‐A species account for most HFMD infections;^2^ however, individual serotypes such as EV‐D68 predominantly cause respiratory tract infections.^3^ RV serotypes are often associated with mild acute upper respiratory tract infections and can be transmitted via aerosol and direct contact.^4^ EV and RV serotypes are typically distinguished based on amino acid sequence divergence of approximately >10% within the viral capsid protein VP4/2 genes^5^ and >13% within the VP1 genes^6^ and have been shown to correlate well with results from virus neutralization assays.^7^

Singapore is an island state in South‐East Asia with a total population of 5.6 million inhabitants and a high burden of respiratory disease and HFMD among other diseases.^8^ HFMD cases among children are notifiable in Singapore;^9^ however, little is known about the prevalence, genetic diversity, and temporal patterns of respiratory EV (including epidemic serotypes such as EV‐D68) and RV among the adult population in Singapore.^10^ To fill this gap, we retrospectively screened 2950 nasal swab samples collected from febrile adults as part of the Early DENgue infection and outcome (EDEN) study, but were dengue virus negative by PCR testing of blood samples collected on the same occasion, during November 2007‐October 2013 for RV/EV serotypes.^11^

## 2 METHODS

Total nucleic acid was extracted from 2950 nasopharyngeal samples sourced from 2091 individuals enrolled in the previously conducted EDEN study^11^ and tested using the Luminex NxTAG Respiratory Pathogen Panel (RPP) kit (Luminex Molecular Diagnostics, Inc). Samples collected at multiple time points were tested for a subset of individuals. The collection of patient clinical and epidemiological data was approved by the National Healthcare Group Institutional Review Board (reference code DSRB B/05/013), and informed consent was obtained for virus screening.^12^ All methods were in accordance with relevant guidelines and regulations; protocols were approved by Institutional Review Board of the National University of Singapore (reference code B‐14‐209E). After reverse transcription and PCR amplification, fluorescence was measured on a MAGPIX multiplex reader and expressed as multi‐dimension detection (MDD) values. The cutoff of 40 MDD pre‐set by the manufacturer was confirmed with positive controls for RV and EV in an in‐house qPCR assay described previously.^13^ Furthermore, the sensitivity of detection of RV/EV RNA in the RPP kit was assessed by and compared to in‐house and commercially available RT‐qPCR assays. The limit of detection for Luminex RPP and in‐house assays was calculated based on dilution series of the positive controls and was found to be within 10‐fold. Whereas all ambiguous values were confirmed with qPCR assays, a limited number of false‐negative detections cannot be ruled out. The genomic region spanning the 5′ UTR and VP4/2 region was amplified using a one‐step PCR (SuperScript III with Platinum Taq High Fidelity Polymerase, Thermo Fisher Scientific) at cycling conditions 55°C, 30 minutes; 94°C, 2 minutes; 94°C, 15 seconds; 45.8‐56.9°C depending on the primer (Table S1), 30 seconds; 68°C, 30 seconds for 40 cycles; 68°C, 5 minutes; 4°C hold, and amplicons (ranging from 506 bp to 1322 bp) were sent to commercial service providers for dideoxy Sanger sequencing. Nucleotide sequences generated in this study were submitted to GenBank (Table S2: MH645807 to MH645811, MH648006 to MH648140, and MH718991).

Multiple sequence alignments were automated using MAFFT v.7^14^ followed by manual optimization and were screened for recombination using the Recombination Detection Program v4.46.^15^ The genetic diversity of RV/EV viruses was determined through phylogenetic analysis of each species based on the combined 5′ UTR and VP4/2 regions including reference sequences of known serotypes. Phylogenies were reconstructed using the maximum likelihood (ML) method in RAxML v8.0,^16^ using the general time‐reversible nucleotide substitution model with a gamma distribution of among‐site rate variation (GTR + G). Node support was estimated with 1000 rapid bootstrap replicates. Trees were visualized and annotated using FigTree v1.4 (http://tree.bio.ed.ac.uk/software/figtree/).

## 3 RESULTS AND DISCUSSION

Eight percent (n = 236) of nasal swab samples collected between 2007 and 2013 from 203 febrile adults in Singapore tested positive for RV or EV as tested using Luminex RPP and qPCR assays (median age 31 years, range 18 to 77, male‐to‐female ratio 2.1:1). This included 13 co‐infections with EV/RV and non‐EV/RV respiratory viruses that were not considered for sequence generation. Thirty‐three samples that resulted in poor sequence reads and 27 that resulted in sequences for only the 5′ UTR were excluded from subsequent analyses. The 5′ UTR and VP4/VP2 regions were generated for the remaining 163 samples that represented 141 patients (median age 30 years, range 18 to 77, male‐to‐female ratio 1.9:1). Samples collected from 22 patients were positive for RV/EV on multiple sampling occasions (average sampling interval of 3.3 days) and presented with the same virus subtype with high genetic similarity on both sampling occasions.

**Figure 1:**
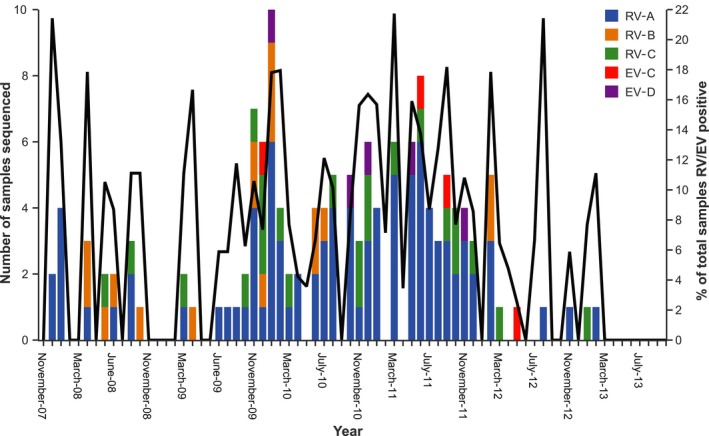
Prevalence of enteroviruses in adults presenting febrile illness in Singapore, 2007‐2013. The proportion of enterovirus/rhinovirus‐positive samples from the total number of samples tested is represented by a solid black line and the number of samples sequenced each month are broken down by species

Rhinoviruses‐A was detected in the majority of patients (92/141, 65.2%) (median age 31 years, range 18‐77, male‐to‐female ratio 1.8:1) and was found in each year between 2007 and 2013, while 25.8% (23/89; median age 27 years, range 18 to 59, male‐to‐female ratio 1.4:1) were RV‐C positive in years 2008‐2010 and 2012, and 12.3% (17/138; median age 29 years, range 18‐72, male‐to‐female ratio 2.8:1) were RV‐B in years 2008‐2013. Subtypes EV‐C (4/141, 2.8%; median age 41 years, range 24‐51, male‐to‐female ratio 3:1) and EV‐D (5/141, 3.5%; median age 26 years, range 24‐46, male‐to‐female ratio 4:1) were only detected sporadically (Figure 1). During 2010 and 2011, our sampling increased substantially (679 samples in 2010 and 573 samples in 2011) compared to other years (2007:12 samples; 2008:195 samples; 2009:352 samples; 2012:282 samples; and 2013:36 samples), leading to a wider range of RV/EV serotypes that were detected in both years, although RV‐B was not detected in 2011. The higher prevalence of RV‐A in Singapore is similar to reports of asymptomatic and hospitalized adults in other regions.^17^ We also found an elevated number of RV/EV detections from December to February and May to July during our surveillance period.

**Figure 2:**
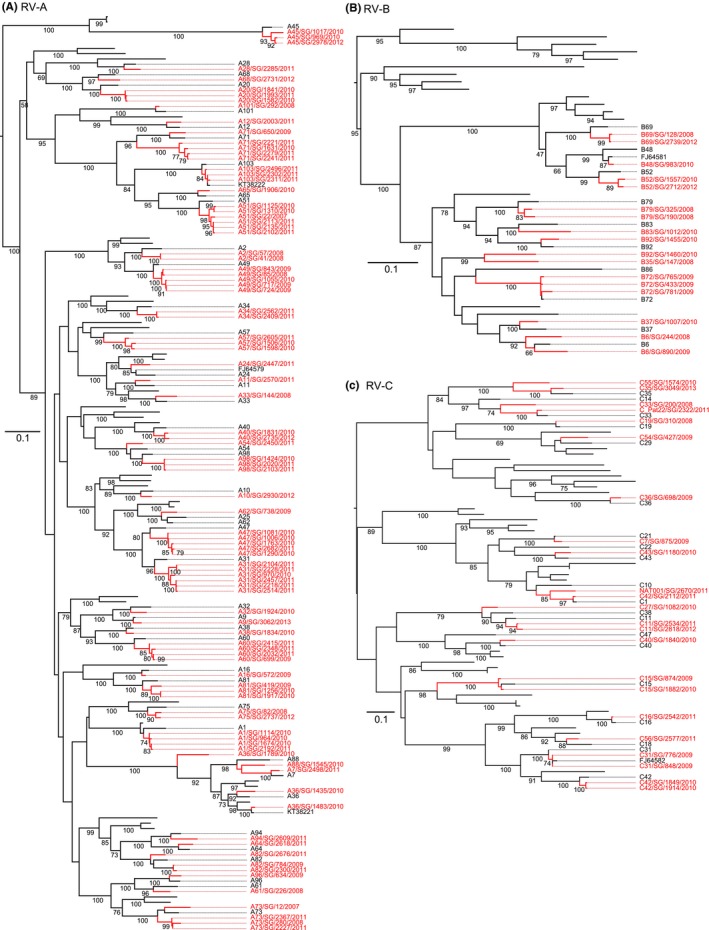
Maximum likelihood phylogenetic trees of the 5′ UTR and VP4/2 regions of RV. Maximum likelihood phylogenies of species RV‐A (A), RV‐B (B), and RV‐C (C). Strains deduced in this study along with the month and year of sampling are provided in red, whereas previously detected RV/EV sequences from Singapore are provided in black along with their GenBank accession number, and the reference strains of each of the serotypes are in black. Scale bars represents nucleotide substitutions per site. Bootstrap values > 70 are shown

The 141 sequences originating from individual patients formed well‐supported clades with reference viruses from 74 serotypes (Figure 2, Figure S1), although there was considerable diversity between the reference strain and sequences generated in this study, indicating within serotype divergence. Serotypes detected in our study included 43 of the 82 known RV‐A serotypes, 10 of the 27 recognized RV‐B serotypes, and 19 of 67 RV‐C, revealing a high serotypic diversity of RV‐A, RV‐B, and RV‐C serotypes in febrile adults in Singapore, whereas the few sporadically detected EV‐C and EV‐D viruses were EV‐C A21 (formerly Coxsackievirus A21) and EV‐D68, respectively (Figure 1, Figure S1). Both EV serotypes are predominantly associated with mild respiratory distress and have been reported to co‐circulate with RV viruses.^3^, ^18^

To identify the potential source populations of viruses sequenced in this study and to better understand the geographical distributions of RV/EV viruses, we conducted large‐scale phylogenetic analysis separately for each RV/EV species using all globally available VP4/2 sequence data. The EV‐D68 phylogeny (Figure S1A) indicates that the four Singaporean D68 samples were distantly related and belong to three distinct subclades (A, B2, and D). Subclade B2 contains viruses predominantly isolated in the Philippines during 2008‐2011, Vietnam in 2009, and Cambodia in 2011, and was divergent to all other clade B2 strains from Europe since 2008 and which appear to be introduced into the United States more recently (Figure S1B), suggesting regional patterns of circulation of this lineage. The single clade A sequence from December 2012 was identical to a strain collected in the UK during the same period; however, the clustering of globally sampled clade A sequences suggests global circulation of clade A during this period. Three clade D sequences collected during 2010 and 2011 formed ancestral relationships to sequences generated from EV‐D68 patients in China, Malaysia, Philippines, and more recently in Cuba and Germany. Taken together, these results suggest frequent migration and introduction of a diverse population of respiratory enterovirus serotypes into Singapore; however, our sampling was insufficient to confirm sustained transmission of these serotypes. Due to low numbers of globally available RV‐A and RV‐B sequence data, no clear geographical or temporal pattern of circulation could be deduced for these serotypes; however, Singaporean RV‐C and EV‐C A21 strains appear to cluster with contemporary isolates collected globally but with little information to delineate region in either of the serotypes (Figure 2C, Figure S1). For example, EV‐C A21 viruses were closely related to the few available sequences of contemporary viruses collected during 2012 to 2016 from China, the Netherlands, and the United States, but there was a gap in sequence data from 2006 to 2012 to identify potential source (Figure S1A).

The pattern of genetic diversity described here (ie, the detection of a high number of RV/EV serotypes and a low number of samples within each serotype) is consistent with studies in other demographics using a variety of diagnostic methodologies. The detection of a low number of samples per serotype in these studies has been a major challenge to the inference of transmission patterns or geographical spread of individual respiratory RV/EV serotypes, except for the epidemic serotype EV‐D68 where substantial genetic information is available. These results fill the gap in our knowledge about the diversity of respiratory EV and RV in febrile adults and highlight the need for improved RV/EV surveillance to aid in the early detection of epidemic strains.

## CONFLICT OF INTEREST

All authors report no potential conflicts of interest.
