# Supplementary material for: Genetic diversity of respiratory enteroviruses and rhinoviruses in febrile adults, Singapore, 2007‐2013
Source: Influenza Other Respir Viruses. 2019 Sep 30;14(1):67–71. doi: 10.1111/irv.12662 (PMC6928033; doi:10.1111/irv.12662)
Supplement: Supplementary file 1 [file IRV-14-67-s001.pdf]

Table S1. Amplification primers used in this study.

Name	Directionality	Specificity	Sequence	Target Region
EV-5'UTR-F	Forward	EV/RV	CAAGCACTTCTGTTTCCC	5'UTR
EV-5'UTR-R	Reverse	EV/RV	CACGGACACCCAAAGTAG	5'UTR
RV-A-1-F	Forward	RV-A	TCCTCCGGCCCCTGAATGYGG	VP4/VP2
RV-B-483-F	Forward	RV-B	TCCTCCGGCCCCTGAATG	VP4/VP2
RV-C-514-F	Forward	RV-C	TCCTCCGGCCCCTGAATG	VP4/VP2
EV-C-458-F	Forward	EV-C	GCCCCTGAATGCGGCTAATC	VP4/VP2
EV-D-436-F	Forward	EV-D	ACTATAGAGTCCTCCGGCCC	VP4/VP2
EV-D-453-F	Forward	EV-D	CCCCTGAATGCGGCTAATCT	VP4/VP2
RV-A-1071-R	Reverse	RV-A	AGTTTCCACCACCAACCTTT	VP4/VP2
RV-A-1077-R	Reverse	RV-A	TTCCACCACCATCCCTTGG	VP4/VP2
RV-A-1081-R	Reverse	RV-A	TCAGGCAATTTCCACCACCA	VP4/VP2
RV-A-1083-R	Reverse	RV-A	TCTGGTAATTTCCACCACCA	VP4/VP2
RV-A-1180-R	Reverse	RV-A	GCAACAACTAATGCACCTTG	VP4/VP2
RV-A-1191-R	Reverse	RV-A	TCTGGTATRGCAGCCACTA	VP4/VP2
RV-A-1214-R	Reverse	RV-A	TGTTCTGGRATAGCAGCAACT	VP4/VP2
RV-A-1240-R	Reverse	RV-A	TCCCACTGTGACATTGCCAG	VP4/VP2
RV-A-1249-R	Reverse	RV-A	GTGTGTATTTGTAACCTGC	VP4/VP2
RV-A-1255-R	Reverse	RV-A	TCACCAGGGTGTGTRTARTT	VP4/VP2
RV-A-1453-R	Reverse	RV-A	GAATCCATRGGAACTGCATT	VP4/VP2
RV-A-1570-R	Reverse	RV-A	AATTCAGCACACATTGGACT	VP4/VP2
RV-A-1586-R	Reverse	RV-A	CACGTGCCCCAGAGAATT	VP4/VP2
RV-A-794-R	Reverse	RV-A	CAGTKGCATCTTGTGGTGTTA	VP4/VP2
RV-A-897-R	Reverse	RV-A	TCAGGYAGTTTCCACCACCA	VP4/VP2
RV-A-929-R	Reverse	RV-A	ATGATGGCCAMACACCATA	VP4/VP2
RV-A-964-R	Reverse	RV-A	CTGTATAGCCACTYCTTCC	VP4/VP2
RV-A-12-R	Reverse	RV-A	GCATCHGGTARTTTCCACCACCA	VP4/VP2
RV-A-1-R	Reverse	RV-A	TCAGGTAATTTCCACCACCA	VP4/VP2
RV-A-12-R	Reverse	RV-A	TTCCACCACCARCCAGATGA	VP4/VP2
RV-A-14-R	Reverse	RV-A	GCATCWGGTARTTTCCACCACCA	VP4/VP2
RV-A-19-R	Reverse	RV-A	TTCCACCACCAYCCTTTTGA	VP4/VP2
RV-A-1041-R	Reverse	RV-A	CCACCACCAGCCTTTTGA	VP4/VP2
RV-A-1172-R	Reverse	RV-A	ATTWAGCCAGTTGTCATCAC	VP4/VP2
RV-B-1692-R	Reverse	RV-B	GGAGAYTGTCTRTCATCTGT	VP4/VP2
RV-B-1097-R	Reverse	RV-B	GCATCTGGWAGYTTCCAACACC	VP4/VP2
RV-C-1125-R	Reverse	RV-C	CCTGCTCTWCCCATTGAGTG	VP4/VP2
RV-C-1137-R	Reverse	RV-C	CTGTGGAATTTAGTRGCATTGC	VP4/VP2
RV-C-1314-R	Reverse	RV-C	AGTGGGTCTTCAGCAGGTTG	VP4/VP2
RV-C-1435-R	Reverse	RV-C	TCCATGGGAACACAGTTTAT	VP4/VP2
RV-C-10-R	Reverse	RV-C	TCTGGTATTTTCCACCACCA	VP4/VP2
RV-C-9-R	Reverse	RV-C	TCAGGYAAYTTCCACCACCA	VP4/VP2
RV-C-4-R	Reverse	RV-C	TCTGGCAGCTTCCACCACCA	VP4/VP2
RV-C-10-R	Reverse	RV-C	CATCYTCYGTTGTCATGAA	VP4/VP2
RV-C-3-R	Reverse	RV-C	TCAGGCAGTTTCCACCACCA	VP4/VP2
RV-C-7-R	Reverse	RV-C	TCTGGAATTTTCCACCACCA	VP4/VP2
RV-C-1071-R	Reverse	RV-C	TTCCACCACCATCCCYTWGA	VP4/VP2
RV-C-1768-R	Reverse	RV-C	GAATCCACTCTWGCCATGTCCA	VP4/VP2
EV-C-1629-R	Reverse	EV-C	CCCCAGTTATTGTGTTTTGC	VP4/VP2
EV-D-1306-R	Reverse	EV-D	GCTCCCCTCTGATGTTCTGG	VP4/VP2
EV-D-1338-R	Reverse	EV-D	TGGCGTTACACTGCACATGA	VP4/VP2

Table S2. Nucleotide length and GenBank accession numbers of viruses detected in this study.

virus name	sequence length	accession number
Rhinovirus A73/Singapore/12/2007	1053	MH648006
Rhinovirus A51/Singapore/22/2007	859	MH648007
Rhinovirus A2/Singapore/41/2008	1216	MH648008
Rhinovirus A2/Singapore/57/2008	1236	MH648009
Rhinovirus A75/Singapore/82/2008	785	MH648010
Rhinovirus A49/Singapore/85/2008	1215	MH648011
Rhinovirus A33/Singapore/144/2008	882	MH648012
Rhinovirus A61/Singapore/226/2008	897	MH648013
Rhinovirus A73/Singapore/280/2008	881	MH648014
Rhinovirus A101/Singapore/292/2008	942	MH648015
Rhinovirus A81/Singapore/419/2009	1047	MH648016
Rhinovirus A16/Singapore/572/2009	1068	MH648017
Rhinovirus A71/Singapore/650/2009	904	MH648018
Rhinovirus A96/Singapore/634/2009	889	MH648019
Rhinovirus A62/Singapore/738/2009	1320	MH648020
Rhinovirus A60/Singapore/699/2009	887	MH648021
Rhinovirus A49/Singapore/717/2009	907	MH648022
Rhinovirus A49/Singapore/724/2009	1249	MH648023
Rhinovirus A49/Singapore/843/2009	1232	MH648024
Rhinovirus A82/Singapore/784/2009	1054	MH648025
Rhinovirus A47/Singapore/1006/2010	1363	MH648026
Rhinovirus A1/Singapore/964/2010	908	MH648027
Rhinovirus A31/Singapore/970/2010	1367	MH648028
Rhinovirus A45/Singapore/969/2010	901	MH648029
Rhinovirus A45/Singapore/1017/2010	905	MH648030
Rhinovirus A47/Singapore/1081/2010	1377	MH648031
Rhinovirus A1/Singapore/1114/2010	908	MH648032
Rhinovirus A51/Singapore/1125/2010	853	MH648033
Rhinovirus A49/Singapore/1055/2010	906	MH648034
Rhinovirus A81/Singapore/1256/2010	1056	MH648035
Rhinovirus A47/Singapore/1290/2010	1403	MH648036
Rhinovirus A51/Singapore/1310/2010	898	MH718991
Rhinovirus A36/Singapore/1483/2010	871	MH648037
Rhinovirus A88/Singapore/1545/2010	848	MH648038
Rhinovirus A98/Singapore/1424/2010	1217	MH648039
Rhinovirus A36/Singapore/1435/2010	1022	MH648040
Rhinovirus A57/Singapore/1506/2010	890	MH648041
Rhinovirus A20/Singapore/1582/2010	864	MH648042
Rhinovirus A57/Singapore/1598/2010	876	MH648043
Rhinovirus A71/Singapore/1631/2010	862	MH648044
Rhinovirus A1/Singapore/1674/2010	907	MH648045
Rhinovirus A36/Singapore/1789/2010	759	MH648046
Rhinovirus A38/Singapore/1834/2010	1363	MH648047
Rhinovirus A47/Singapore/1763/2010	1360	MH648048
Rhinovirus A40/Singapore/1831/2010	1358	MH648049
Rhinovirus A20/Singapore/1841/2010	881	MH648050
Rhinovirus A65/Singapore/1906/2010	897	MH648051
Rhinovirus A81/Singapore/1917/2010	879	MH648052

Rhinovirus A32/Singapore/1924/2010	909	MH648053
Rhinovirus A98/Singapore/2020/2011	1349	MH648054
Rhinovirus A20/Singapore/1993/2011	893	MH648055
Rhinovirus A12/Singapore/2003/2011	1019	MH648056
Rhinovirus A60/Singapore/2032/2011	1085	MH648057
Rhinovirus A31/Singapore/2104/2011	1368	MH648058
Rhinovirus A51/Singapore/2102/2011	848	MH648059
Rhinovirus A51/Singapore/2113/2011	872	MH648060
Rhinovirus A51/Singapore/2135/2011	874	MH648061
Rhinovirus A98/Singapore/2103/2011	1347	MH648062
Rhinovirus A1/Singapore/2192/2011	910	MH648063
Rhinovirus A71/Singapore/2221/2011	864	MH648064
Rhinovirus A73/Singapore/2227/2011	896	MH648065
Rhinovirus A31/Singapore/2228/2011	878	MH648066
Rhinovirus A31/Singapore/2218/2011	1367	MH648067
Rhinovirus A60/Singapore/2348/2011	950	MH648068
Rhinovirus A71/Singapore/2241/2011	872	MH648069
Rhinovirus A71/Singapore/2279/2011	882	MH648070
Rhinovirus A28/Singapore/2285/2011	889	MH648071
Rhinovirus A103/Singapore/2302/2011	871	MH648072
Rhinovirus A82/Singapore/2300/2011	1071	MH648073
Rhinovirus A103/Singapore/2311/2011	882	MH648074
Rhinovirus A73/Singapore/2367/2011	1067	MH648075
Rhinovirus A34/Singapore/2409/2011	876	MH648076
Rhinovirus A60/Singapore/2415/2011	885	MH648077
Rhinovirus A54/Singapore/2450/2011	838	MH648078
Rhinovirus A103/Singapore/2496/2011	890	MH648079
Rhinovirus A34/Singapore/2562/2011	876	MH648080
Rhinovirus A11/Singapore/2570/2011	901	MH648081
Rhinovirus A57/Singapore/2605/2011	878	MH648082
Rhinovirus A24/Singapore/2447/2011	871	MH648083
Rhinovirus A31/Singapore/2457/2011	1378	MH648084
Rhinovirus A7/Singapore/2498/2011	771	MH648085
Rhinovirus A31/Singapore/2514/2011	872	MH648086
Rhinovirus A94/Singapore/2609/2011	1016	MH648087
Rhinovirus A64/Singapore/2618/2011	1018	MH648088
Rhinovirus A68/Singapore/2731/2012	884	MH648089
Rhinovirus A82/Singapore/2676/2011	1094	MH648090
Rhinovirus A47/Singapore/2682/2011	1370	MH648091
Rhinovirus A40/Singapore/2735/2012	1351	MH648092
Rhinovirus A75/Singapore/2737/2012	901	MH648093
Rhinovirus A10/Singapore/2930/2012	1371	MH648094
Rhinovirus A45/Singapore/2978/2012	901	MH648095
Rhinovirus A9/Singapore/3062/2013	882	MH648096
Rhinovirus B69/Singapore/128/2008	887	MH648097
Rhinovirus B35/Singapore/147/2008	840	MH648098
Rhinovirus B79/Singapore/190/2008	1468	MH648099
Rhinovirus B6/Singapore/244/2008	901	MH648100
Rhinovirus B79/Singapore/325/2008	1285	MH648101
Rhinovirus B72/Singapore/433/2009	900	MH648102
Rhinovirus B72/Singapore/765/2009	900	MH648103

Rhinovirus B72/Singapore/781/2009	887	MH648104
Rhinovirus B83/Singapore/1012/2010	1394	MH648105
Rhinovirus B37/Singapore/1007/2010	892	MH648106
Rhinovirus B6/Singapore/890/2009	889	MH648107
Rhinovirus B48/Singapore/983/2010	898	MH648108
Rhinovirus B92/Singapore/1460/2010	898	MH648109
Rhinovirus B92/Singapore/1455/2010	1337	MH648110
Rhinovirus B52/Singapore/1577/2010	890	MH648111
Rhinovirus B69/Singapore/2739/2012	884	MH648112
Rhinovirus B52/Singapore/2712/2012	890	MH648113
Rhinovirus C33/Singapore/200/2008	1002	MH648114
Rhinovirus C19/Singapore/310/2008	860	MH648115
Rhinovirus C54/Singapore/427/2009	871	MH648116
Rhinovirus C36/Singapore/698/2009	862	MH648117
Rhinovirus C31/Singapore/776/2009	959	MH648118
Rhinovirus C31/Singapore/848/2009	885	MH648119
Rhinovirus C15/Singapore/874/2009	878	MH648120
Rhinovirus C7/Singapore/875/2009	1532	MH648121
Rhinovirus C27/Singapore/1082/2010	895	MH648122
Rhinovirus C43/Singapore/1180/2010	1519	MH648123
Rhinovirus C55/Singapore/1574/2010	937	MH648124
Rhinovirus C40/Singapore/1840/2010	879	MH648125
Rhinovirus C42/Singapore/1849/2010	908	MH648126
Rhinovirus C15/Singapore/1882/2010	887	MH648127
Rhinovirus C42/Singapore/1914/2010	881	MH648128
Rhinovirus C42/Singapore/2112/2011	890	MH648129
Rhinovirus C_Pat22/Singapore/2322/2011	1134	MH648130
Rhinovirus C11/Singapore/2534/2011	898	MH648131
Rhinovirus C16/Singapore/2542/2011	893	MH648132
Rhinovirus C56/Singapore/2577/2011	885	MH648133
Rhinovirus NAT001/Singapore/2670/2011	1440	MH648134
Rhinovirus C11/Singapore/2818/2012	1209	MH648135
Rhinovirus C35/Singapore/3049/2008	994	MH648136
Human coxsackievirus A21/Singapore/1890/2010	1361	MH648137
Human coxsackievirus A21/Singapore/2314/2011	1377	MH648138
Human coxsackievirus A21/Singapore/2481/2011	1390	MH648139
Human coxsackievirus A21/Singapore/2871/2012	1377	MH648140
Enterovirus D68/Singapore/1004/2010	1066	MH645807
Enterovirus D68/Singapore/1823/2010	1057	MH645808
Enterovirus D68/Singapore/1936/2010	1059	MH645809
Enterovirus D68/Singapore/2203/2011	1042	MH645810
Enterovirus D68/Singapore/2614/2011	1074	MH645811

a

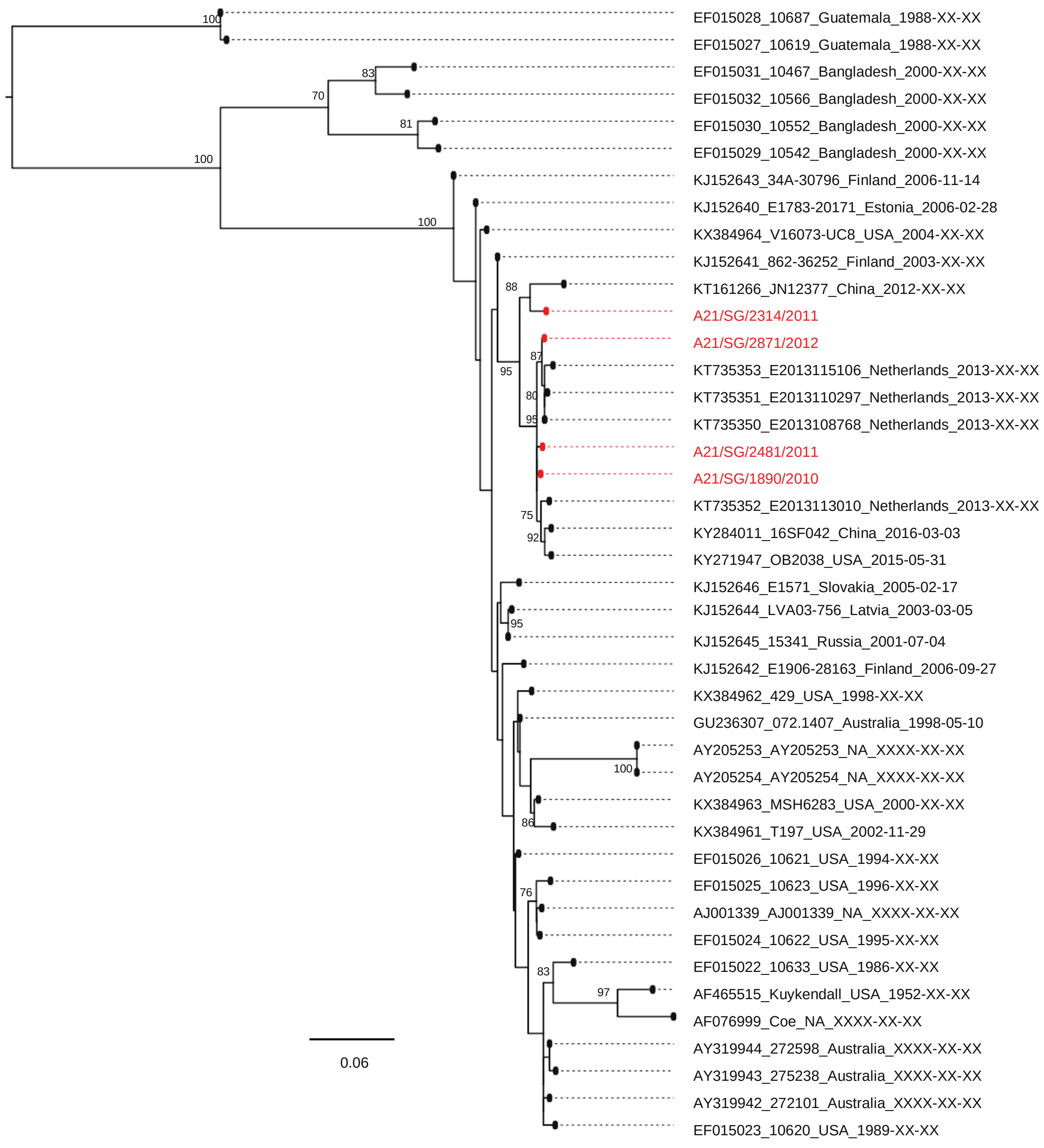

EF015028_10687_Guatemala_1988-XX-XX
EF015027_10619_Guatemala_1988-XX-XX
EF015031_10467_Bangladesh_2000-XX-XX
EF015032_10566_Bangladesh_2000-XX-XX
EF015030_10552_Bangladesh_2000-XX-XX
EF015029_10542_Bangladesh_2000-XX-XX
KJ152643_34A-30796_Finland_2006-11-14
KJ152640_E1783-20171_Estonia_2006-02-28
KX384964_V16073-UC8_USA_2004-XX-XX
KJ152641_862-36252_Finland_2003-XX-XX
KT161266_JN12377_China_2012-XX-XX
A21/SG/2314/2011
A21/SG/2871/2012
KT735353_E2013115106_Netherlands_2013-XX-XX
KT735351_E2013110297_Netherlands_2013-XX-XX
KT735350_E2013108768_Netherlands_2013-XX-XX
A21/SG/2481/2011
A21/SG/1890/2010
KT735352_E2013113010_Netherlands_2013-XX-XX
KY284011_16SF042_China_2016-03-03
KY271947_OB2038_USA_2015-05-31
KJ152646_E1571_Slovakia_2005-02-17
KJ152644_LVA03-756_Latvia_2003-03-05
KJ152645_15341_Russia_2001-07-04
KJ152642_E1906-28163_Finland_2006-09-27
KX384962_429_USA_1998-XX-XX
GU236307_072.1407_Australia_1998-05-10
AY205253_AY205253_NA_XXXX-XX-XX
AY205254_AY205254_NA_XXXX-XX-XX
KX384963_MSH6283_USA_2000-XX-XX
KX384961_T197_USA_2002-11-29
EF015026_10621_USA_1994-XX-XX
EF015025_10623_USA_1996-XX-XX
AJ001339_AJ001339_NA_XXXX-XX-XX
EF015024_10622_USA_1995-XX-XX
EF015022_10633_USA_1986-XX-XX
AF465515_Kuykendall_USA_1952-XX-XX
AF076999_Coe_NA_XXXX-XX-XX
AY319944_272598_Australia_XXXX-XX-XX
AY319943_275238_Australia_XXXX-XX-XX
AY319942_272101_Australia_XXXX-XX-XX
EF015023_10620_USA_1989-XX-XX
0.06

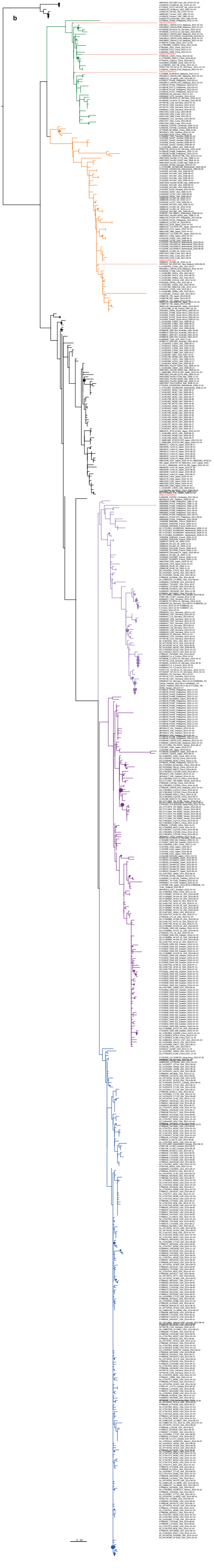
b

Figure S1. Maximum likelihood phylogenies of the 5' UTR and VP4/2 regions of EV-C A21 (a) and EV-D68 (b) showing potential source populations of Singaporean strains. Singapore strains along with the month and year of sampling are provided in red, and the reference strains of each of the serotypes are in black. Scale bar represents nucleotide substitutions per site. >70 bootstrap values are shown.
